# Suicidal Behavior in Transgender and Non-Binary Individuals: Association of Depressive Symptoms, Loneliness, and Childhood Trauma

**DOI:** 10.1192/j.eurpsy.2026.12074

**Published:** 2026-07-31

**Authors:** I. D. F. P. Cahet, M. L. Medeiros, V. Leao De Melo Neto

**Affiliations:** Faculty of Medicine, Federal University of Alagoas, Maceió, Brazil

## Abstract

**Introduction:**

According to the World Health Organization, over 700,000 people die by suicide annually worldwide, making it the third leading cause of death for both sexes in the 15-29 age group. Social minority groups, notably transgender and non-binary individuals, are predisposed to a higher incidence of suicidal behavior possibly due to their increased feelings of loneliness, high rates of physical, and sexual violence, bullying, and discrimination.

**Objectives:**

To investigate the association between suicidal behavior and risk factors, such as depressive symptoms, perception of loneliness, and history of childhood and adolescent trauma, in transgender and non-binary individuals.

**Methods:**

This analytical cross-sectional study involved the self-completion of an online questionnaire covering sociodemographic aspects, depression (PHQ-9 – Patient Health Questionnaire), feelings of loneliness (UCLA – UCLA Loneliness Scale, Brazilian Version 3), early trauma (CTQ – Childhood Trauma Questionnaire), and suicidal behavior (Mini International Neuropsychiatric Interview, 7.2) by Brazilian binary and non-binary trans individuals aged over 18. For statistical analysis, participants were divided into two groups according to the severity of suicidal behavior and three according to gender identity. Inferential analysis involved non-parametric tests and the chi-square test.

**Results:**

Among the 285 participants, 127 were trans men, 90 were trans women, and 68 were non-binary people. Of the total sample, 70.9% were aged 18-29, 40.7% were Afro-descendants, 81.1% were single, 47% had up to basic education, 44.2% were unemployed, and 83.9% had no religious affiliation. Among all participants, 83.2% presented moderate to severe depression, 80% experienced moderate to severe loneliness, 53% reported emotional abuse, and 49.9% reported a history of sexual abuse. The prevalence of clinically significant suicidal behavior (moderate or severe risk) was 75.8% of the total sample. Moderate to severe suicidal behavior was more prevalent in young people aged 18-29 (OR=2.37; 95% CI=1.34-4.17; p=0.036). It was significantly associated with moderate to severe depressive symptoms (OR= 18.54; 95% CI=8.5-40.39; p<0.001), emotional abuse (OR= 2.91; 95% CI= 1.64-5.14; p<0.001), and sexual abuse (OR= 2.26; 95% CI= 1.29-3.97; p=0.008). The main protective factors identified were: higher education (p=0.009), being engaged in the labor market (p=0.003), undergoing hormone therapy (p<0.001), and absence of comorbidities (p<0.001).

**Conclusions:**

The sample exhibits a high prevalence of depression, loneliness, early trauma, and suicidal behavior. Participants with clinically significant suicidal behavior presented greater severity of depressive symptoms, as well as a higher history of emotional and sexual abuse.

**Disclosure of Interest:**

None Declared

